# Competition from sea to mountain: Interactions and aggregation in low‐diversity monogenean and endohelminth communities in twospot livebearer *Pseudoxiphophorus bimaculatus* (Teleostei: Poeciliidae) populations in a neotropical river

**DOI:** 10.1002/ece3.6557

**Published:** 2020-08-12

**Authors:** Guillermo Salgado‐Maldonado, Juan Manuel Caspeta‐Mandujano, Edgar F. Mendoza‐Franco, Miguel Rubio‐Godoy, Adriana García‐Vásquez, Norman Mercado‐Silva, Ismael Guzmán‐Valdivieso, Wilfredo A. Matamoros

**Affiliations:** ^1^ Instituto de Biología Universidad Nacional Autónoma de México Ciudad de México Mexico; ^2^ Facultad de Ciencias Biológicas Universidad Autónoma del Estado de Morelos Cuernavaca Mexico; ^3^ EPOMEX Universidad Autónoma de Campeche Campeche Mexico; ^4^ Instituto de Ecología Xalapa México; ^5^ Centro de Investigación en Biodiversidad y Conservación Universidad Autónoma del Estado de Morelos Cuernavaca Mexico; ^6^ Instituto de Ciencias Biológicas Universidad de Ciencias y Artes de Chiapas Chiapas Mexico

**Keywords:** assembly, competition, interspecific aggregation, intraspecific aggregation, repeatability of community structure, species coexistence, species richness, species saturation of communities

## Abstract

1. The role of interspecific interactions in structuring low‐diversity helminth communities is a controversial topic in parasite ecology research. Most parasitic communities of fish are species‐poor; thus, interspecific interactions are believed to be unimportant in structuring these communities.
2. We explored the factors that might contribute to the richness and coexistence of helminth parasites of a poeciliid fish in a neotropical river.
3. Repeatability of community structure was examined in parasitic communities among 11 populations of twospot livebearer *Pseudoxiphophorus bimaculatus* in the La Antigua River basin, Veracruz, Mexico. We examined the species saturation of parasitic communities and explored the patterns of species co‐occurrence. We also quantified the associations between parasitic species pairs and analyzed the correlations between helminth species abundance to look for repeated patterns among the study populations.
4. Our results suggest that interspecific competition could occur in species‐poor communities, aggregation plays a role in determining local richness, and intraspecific aggregation allows the coexistence of species by reducing the overall intensity of interspecific competition.

## Graphical abstract

The results of this work suggest that interspecific competition can occur in species‐poor, nonsaturated communities among specialist dominant species. Our analyses suggest also that aggregation is an important factor to determine local richness of parasites in fish populations and that intraspecific aggregation allows the coexistence of species in the same host population by means of reducing the overall intensity of competition.

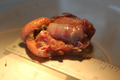

## 1 INTRODUCTION

Parasitic systems enable us to explore essential aspects of ecology (Poulin, 2007; Poulin & Morand, 2004). It is important to understand how local communities are configured and the interactions among species within a region. Holmes and Price (1986) recognized both interactive and isolationist parasitic communities. In the former, between‐species interactions are important for structuring the community. In the latter, interspecies interactions play nondetectable roles and the influence of competition is negligible (Poulin, 2007). Thus, the presence of any species is independent of the presence of other species in isolationist communities (Price, 1980; Rohde, 1979). In species‐rich parasitic communities that have high abundances, such as bird communities, both interspecific and intraspecific interactions among parasites are important forces that structure the community (e.g., Bush & Holmes, 1986; Stock & Holmes, 1988). In some fishes, mostly elasmobranchs, rich helminth communities have been described and interactions between species have been documented (e.g., Agrawal, Rajvanshi, & Asthana, 2017; Randhawa, 2012).

The importance of interspecific interactions for community structuring in low‐diversity helminth communities remains a controversial topic. Caswell (1976) suggested that noninteractive communities lack saturation and species can coexist in the community because space is not a limiting factor. Most parasitic communities of fish are species‐poor and unsaturated with species; therefore, interspecific interactions are not important in structuring such communities (e.g., Gotelli & Rohde, 2002; Muñoz, Mouillot, & Poulin, 2006). Low‐diversity parasitic assemblages are mostly structured by intraspecific, rather than interspecific, interactions (e.g., Haukisalmi & Henttonen, 1993; Morand, Poulin, Rohde, & Hayward, 1999). However, Kennedy (1992) suggested that interspecific competition can occur in species‐poor isolationist communities. Additional empirical support by Vidal‐Martínez and Kennedy (2000) showed that even relatively small numbers of acanthocephalans can produce a displacement of phylogenetically unrelated intestinal helminths (trematodes and nematodes) in a tropical cichlid fish, *Cichlasoma synspilum*. We recently described the potential interactions in low‐diversity monogenean parasitic communities in a tropical freshwater fish, *Astyanax aeneus*, and showed that interspecific interactions can be an important factor for structuring low‐diversity ectoparasitic helminth communities (Salgado‐Maldonado, Mendoza‐Franco, Caspeta‐Mandujano, & Ramírez‐Martínez, 2019). Thus, the extent to which interspecific interactions are important structuring factors for low‐diversity fish ecto‐ and endoparasite communities remains uncertain.

Interspecific interactions may lead to species exclusion; however, there are several ways in which species can coexist (Morand et al., 1999). Aggregated resource use may reduce the overall competition intensity and is key to local parasite (monogeneans) richness in fish populations (Agrawal et al., 2017; Morand et al., 1999; Šimková, Desdevises, Gelnar, & Morand, 2000; Šimková, Gelnar, & Sasal, 2001). Intraspecific aggregation allows the coexistence of species that would otherwise be excluded. More parasitic species can coexist in the same host population when their distributions between individual hosts are aggregated (Ives, 1988, 1991). The host population represents a collection of resource patches among which the parasites are heterogeneously distributed. Some patches (i.e., hosts) harbor many individuals (parasites), whereas others only a few. Aggregation thus refers to the degree to which individuals are added between patches (Ives, 1991). Generally, parasitic populations are distributed in an aggregated manner among individual hosts (i.e., the majority of hosts have a few parasites and most parasites are concentrated in a few hosts; Poulin, 1998a, 1998b; Poulin & Morand, 2004). Aggregation is the most common feature of metazoan parasitic infections (Poulin, 1993; Šimková et al., 2000).

To assess the repeatability of community structure in space, we examined parasitic community organization among 11 populations of twospot livebearer *Pseudoxiphophorus bimaculatus* (Heckel, 1848; Teleostei: Poeciliidae). We examined species richness patterns and whether the parasitic communities were species saturated, and explored patterns of species co‐occurrence. We quantified the associations between parasitic species pairs (e.g., Dezfuli, Giari, De Boaggi, & Poulin, 2001; Haukisalmi & Henttonen, 1993), considering that positive or negative associations between parasitic species suggested a departure from random co‐occurrence (Poulin, 2001, 2007). Further, we analyzed the correlations between the abundance of different helminth species and whether the observed patterns were repeated across the study populations. Our study model consisted of many patches that were identical in resources (hosts) and sustained several helminth populations.

The level of competition that a helminth experiences depends on the number and species of helminths sharing the same patch (host), the distribution of helminths in those patches, and the number of hosts available to invade in each locality, that is, the density of host species. We assessed the level of aggregation of helminth populations to test their influence on determining the local parasite richness within a host population. Further, we assessed whether intraspecific aggregation exceeded interspecific aggregation (e.g., Salgado‐Maldonado et al., 2019). Our goal was to explore the factors that might contribute to the richness and coexistence of helminth parasites of *P*. *bimaculatus* across 11 localities in the La Antigua River, a neotropical system in Veracruz, Mexico. Populations of the poeciliid *P*. *bimaculatus* in the La Antigua River were chosen for the present study because some aspects of their parasite community structure have been described previously, including information on common and rare species (Salgado‐Maldonado et al., 2014); however, information on community saturation, intraspecific and interspecific aggregation, and consistency of pairwise species associations is limited.

## 2 METHODS

### 2.1 Study area

**FIGURE 1:**
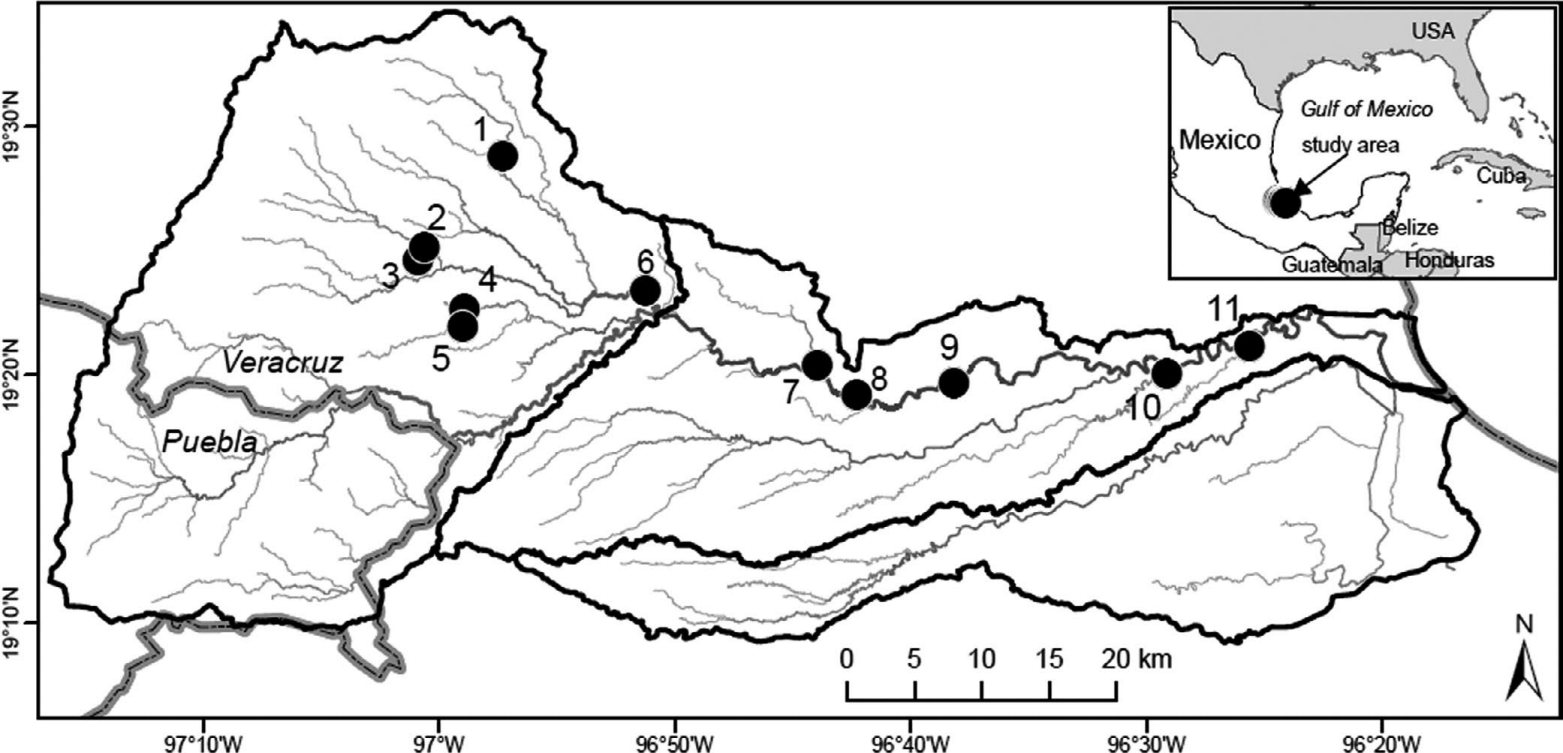
The río La Antigua basin at Veracruz, Mexico. Sampled localities are as follows: 1, Pixquiac (Coord: UTM 14Q 0715115, 2154905; altitude 1,245 m a.s.l.), 2. Xico (0709328, 2148062; 1,438 m), 3. Agua Bendita (0708849, 2147130; 1,278 m), 4. Teocelo (0712295, 2143510; 1,115 m), 5. Baxtla (0712154, 2142160; 1,105 m), 6. Jalcomulco (0725770, 2144871; 617 m), 7. Apazapan (0738583, 2139350; 328 m), 8. Río de Los Pescados (0741490, 2137128; 282 m), 9. El Carrizal (0748702, 2138013; 211 m), 10. Puente Nacional (0764574, 2138651; 78 m), and 11. Antigua Presa (770702, 2140755; 42 m)

The study was conducted at 11 sites located between 42 and 1,245 m above sea level (a.s.l.) within the La Antigua River basin (Figure 1). The La Antigua River is a high‐gradient foothill river originating from the Cofre de Perote volcano and adjacent mountains from the Sierra Madre Oriental (altitude 4,200 m) in the states of Puebla and Veracruz, Mexico. Typical of rivers in hilly terrain, numerous headwater streams combine to form montane and piedmont canyons. The upper watershed of the La Antigua River covers a wide altitudinal range, from 480 to 4,200 m a. s. l. before the river arrives at the coastal plain. The river runs approximately 100 km east of the Gulf of Mexico (Mercado‐Silva, Lyons, Díaz‐Pardo, Navarrete, & Gutiérrez‐Hernández, 2012).

### 2.2 Host species and sampling

*Pseudoxiphophorus* *bimaculatus* is distributed on the Atlantic slope, from the Misantla River, Veracruz, Mexico, southward to the Nombre de Dios ichthyo‐province on the Caribbean side of Honduras (Matamoros, Schaefer, & Kreiser, 2009). The species prefers well‐shaded, slowly moving, fairly deep (up to 1.3 m) waters with fallen leaves and brush piles or overhanging riparian plants in creeks, lagoons, rivers, and swampy pools containing a variety of substrates. It feeds mainly on Culicidae (Diptera; Trujillo‐Jiménez & Toledo, 2007) and attains a maximum total length of 80 mm (Miller, Minckley, & Norris, 2005).

We examined 19 *P*. *bimaculatus* from Agua Bendita, 21 from Puente Nacional, and 20 from each of the other nine locations sampled in June 2016 (Figure 1). Specimens were collected under collecting permit FAUT‐0105. Fish were collected using DC backpack electroshockers, seines, and gill nets. Captured individuals were placed in plastic bags filled with water, transferred to the laboratory, and kept alive in aerated containers until subsequent examination for the presence of helminth parasites (within 8 hr of capture). To complete the examination, fish were euthanized with an overdose of the anesthetic 2‐phenoxyethanol (Sigma‐Aldrich, St. Louis, Missouri), measured (total and standard lengths), and examined under a stereomicroscope in Petri dishes containing river water. Externally, the skin, scales, mouth, branchial cavity, anus, and fins of each host were examined. The branchial arches were removed, separated from the branchial cavity, and evaluated individually. All internal tissues, including the digestive tract, body musculature, and organs, were examined for helminth parasites. The helminths that were obtained from the dissections were counted and recorded separately for each fish.

The overall parasite population structure was described using the following parameters as described by Bush, Lafferty, Lotz, and Shostak (1997): prevalence (percent of hosts infected), mean intensity (mean number of helminth individuals of a given species per infected host), and mean abundance (mean number of helminth individuals of a given species per examined host). Analyses were conducted at two hierarchical community levels (Holmes & Price, 1986): (a) infracommunity, which included the parasites of each fish examined, and (b) component community, referring to helminths of all hosts examined at each location and date. Because parasites inhabit different parts of the host and are not in contact with each other, we analyzed ectoparasitic monogeneans and endoparasites separately. Generally, ectoparasitic monogeneans and endoparasites are considered noninteractive communities (Muñoz et al., 2006; however see Larsen, Bresciani, & Buchmann, 2002).

### 2.3 Data analysis

#### 2.3.1 Richness

To assess the effectiveness of our sampling effort, all component communities were evaluated using species accumulation curves. Sampling adequacy for all component communities (i.e., the total number of fish examined from each locality) was evaluated using randomized (100×) sample‐based species accumulation curves computed in EstimateS (version 8.0 RK Coldwell, http://viceroy.eeb.unconn.edu/estimates). For each component, we examined the asymptotic richness based on Clench's model equation (Soberón & Llorente, 1993) as well as the final slope of the randomized species accumulation curve (Jiménez‐Valverde & Hortal, 2003), that is, the gradient between the final two sampling points (see Table 1). A final value of the slope of the species accumulation curve not greater than 0.1 species per sample was used as the criterion for adequate sampling because empirically this final slope indicates that at least 70% of the species in the component community had already been sampled (Jiménez‐Valverde & Hortal, 2003). In addition, we estimated the number of rare species that were not detected in each component community using the nonparametric Bootstrap estimator (Table 1).

**TABLE 1:** Measures, equations, parameters keys, and references used in data analyses

Measure	Equation	Parameter key	Reference
Clench's model	V2=(a∗V1)/(1+(b∗V1))	*V*2 = observed richness *V*1 = number of hosts examined *a* and *b* are parameters of the curve, *a* equals the rate of adding new species, and *b* is a parameter related to the shape of the curve. The slope of the cumulative species curve was calculated as *a*/(1 + *b* * *n*)^2^, where *a* and *b* are the above parameters and *n* is the number of hosts examined from a given component. Clench's model equation allows the estimation of the total number of species in a component as *a*/*b*.	Soberón and Llorente (1993), Jiménez‐Valverde and Hortal (2003)
Bootstrap estimator (*S*_b_)	*S*_b_ = *S*_O_ + Ʃ [1–(*h_j_*/*H*)]^H^	*S*_O_ = observed species richness *H* = number of host individuals sampled from the component community *h_j_* = number of host individuals in the sample in which parasite species *j* is found.	Poulin (1998)
Intraspecific aggregation	$J1=\frac{\sum_{i}^{n}\frac{n1i\left(n1i‐1\right)}{m1}‐m1}{m1}=\frac{\frac{V1}{m1}‐1}{m1}$	*n*_1_ *_i_* = number of helminths of species 1 in the host *i* *m*_1_ = mean number of helminth individuals of species 1 per host, *V*_1_ = variance in the number of helminth species 1.	Ives (1988)
Interspecific aggregation	$C{1,2}^{}=\frac{\sum_{i=1}^{P}\frac{n1in2i}{m1P}‐m2}{m2}=\frac{\text{Cov}1,2}{m1m2}$	*n*_1_ *_i_* and *n*_2_ *_i_* = numbers of helminths of species 1 and 2 in the host *i* *m*_1_ *_i_* and *m*_2_ *_i_* = mean number of helminths per host of species 1 and 2 *P* = number of hosts Cov = covariability between a pair of species.	Ives (1988, 1991)
Decrease in competition	$A{1,2}^{}=\frac{\left(J1+1)(J2+1\right)}{(C{1,2}_{}+1)2^{}}$	All variables apply as described above	Morand et al. (1999)

#### 2.3.2 Saturation

To explore local–regional richness relationships, we plotted the mean infracommunity parasite richness (local richness) versus the component community parasite species richness (regional richness) and calculated the function that best fit the data (Cornell, 1996; Kennedy & Guégan, 1994; Morand et al., 1999; Poulin, 2007). When local richness is regressed against regional richness and the relationship is linear, communities are unsaturated and exhibit proportional sampling of the regional species pool. If the relationship is somewhat curvilinear, the possibility of saturation may occur (Guégan, Morand, & Poulin, 2005). According to Morand et al. (1999), the dependence of infracommunity richness on the component community richness indicates nonsaturation. The maximum observed infracommunity richness was examined because the co‐occurrence of all species found in a component community in a single host individual is unlikely unless their prevalence is very high. A proportional relationship between maximum richness recorded in an infracommunity and the observed richness in the component community suggests that a maximum level of richness does not exist and is consistent with the absence of saturation in the communities (Morand et al., 1999).

#### 2.3.3 Intraspecific aggregation

We quantified the intra‐ and interspecific aggregation of helminths. We calculated the parameter *J* value for each helminth taxon (Table 1) as an intraspecific aggregation measurement that quantified the relative increase in conspecific competitors above the average number that a helminth experiences when infecting a new host. The *J* value is a measure of the proportional increase in the number of conspecific competitors that an individual helminth experiences from a random distribution. A value of *J* = 0 indicates that individual helminths are randomly distributed, whereas a value of *J* = 0.5 indicates a 50% increase in the average number of conspecific helminths expected in the patch (host) above what would be expected if the individuals were randomly distributed (Ives, 1988). In other words, *J* = 0.5 indicates a 50% increase in the aggregation of individuals of the same species in a host (Šimková et al., 2001).

#### 2.3.4 Interspecific aggregation

To measure the association between two species in each of the infracommunities, we calculated the C_1,2_ index (Table 1), which is a measure of the proportional increase in the number of heterospecific helminth competitors regarding a random association. C_1,2_ is the relative change in the average number of heterospecific helminths with which the helminths of species 1 have to compete when species are not independently distributed (Ives, 1988). When *C* > 0, both species are positively correlated and thus aggregated in the host (Ives, 1988). If *C* < 0, species are negatively correlated and there is segregation between species. If *C*_1,2_ = 0.5, there is 50% of the expected number of heterospecific competitors in the host, above what one would expect if helminth species 1 and 2 were randomly distributed (Šimková et al., 2001).

#### 2.3.5 Associations between pairs of parasite species and correlations

The abundance of a parasite species in a host may depend on the presence or abundance of a second species. Identifying patterns of species co‐occurrence and association can provide strong evidence of the importance of positive or negative interspecific interactions in structuring communities (Dezfuli et al., 2001; Poulin, 2001, 2007; Rohde, 1994). Pairwise analyses of species associations allow the identification of nonrandom patterns, with repeatability in space assessed across similar host populations to examine parasite community organization (Poulin & Valtonen, 2002).

The quantification of associations between the pairs of parasite species represents a basic null model approach (Poulin & Valtonen, 2002). No association indicates that two parasite species are randomly distributed among hosts, and a positive or negative association between parasite species suggests a departure from random occurrence (Dezfuli et al., 2001; Poulin, 2001, 2007; Poulin & Valtonen, 2002; Vidal‐Martínez & Kennedy, 2000).

We used Spearman's rank correlation coefficient to evaluate the correlation between the intensities of two helminth species across hosts; we removed fish that were not infected by either of the two parasite species. In all cases, we indicated the statistical significance of Spearman's coefficient values with an asterisk: *p < .05; **p < .01, ***p < .001.

#### 2.3.6 Decrease in competition

To evaluate the decrease in competition owing to intraspecific aggregation, we compared the relative intensity of intraspecific aggregation versus interspecific aggregation in a pair of species, 1 and 2, by calculating *A*_1,2_ (Table 1). If *A*_1,2_ > 1, intraspecific aggregation was greater than interspecific aggregation, and vice versa.

## 3 RESULTS

### 3.1 Community composition

A total of 18 helminth taxa were found in the present study (Table 2). Monogeneans were the most prevalent, abundant, and widely distributed group, being recorded in eight out of the 11 sampling locations. They occupied the highest number of patches (infracommunities and component communities) and were the most numerous parasites in these patches (Table 3). Together, the four species of monogeneans found accounted for 43% (1048/2407) of all helminths collected in the study (Table A1: Appendix S1). One to four species of endohelminths were recorded from seven out of the 11 locations (Tables 3 and Table A1: Appendix S1). The four endohelminths (two adult trematodes and two adult nematodes) accounted for 16% (385/2407) of all helminth individuals. A third group of 10 taxa of helminths, including metacercariae and larval nematodes, accounted for 40% (974/2407) of all helminths. However, metacercariae of *Centrocestus formosanus* recorded from five localities accounted for 83% (811/974) of larval helminths; the remaining nine taxa of this group were mostly rare and scattered in a few locations.

**TABLE 2:** Helminth parasites of *Pseudoxiphophorus bimaculatus* collected in June 2016 from 11 localities of La Antigua river basin, Veracruz, Mexico

Parasite species	Microhabitat
Monogenea	
Dactylogyridae Bychowsky, 1933	
*Urocleidoides vaginoclaustroides* Mendoza‐Franco, Caspeta‐Mandujano, Salgado‐Maldonado and Matamoros, 2015	Gills
Gyrodactylidae van Beneden and Hesse, 1863	
*Gyrodactylus takoke* García‐Vásquez, Razo‐Mendivil and Rubio‐Godoy, 2015	Fins
*G*. *xalapensis* Rubio‐Godoy, Paladini, García‐Vásquez and Shinn, 2010	Fins
*Gyrodactylus* sp.	Fins
Trematoda	
Gorgoderidae Looss, 1901	
*Phyllodistomum inecoli* Razo‐Mendivil, Pérez Ponce de León and Rubio‐Godoy, 2013	Urinary bladder
Allocreadiidae Looss, 1902	
*Paracreptotrematoides heterandriae* (Salgado‐Maldonado, Caspeta‐Mandujano and Vazquez, 2012)	Intestine
Metacercariae	
Echinostomatidae Looss, 1899	
*Echinochasmus leopoldinae* Scholz, Ditrich and Vargas‐Vázquez, 1996	Intestinal mucosa
Heterophyidae Odhner, 1914	
*Centrocestus formosanus* (Nishigori, 1924)	Gills
*Ascocotyle* (*Leighia*) *megalocephala* Price, 1932	Intestinal mucosa
*A*. (*Phagicola*) *macrostoma* (Robinson, 1956)	Gills
Clinostomidae Lühe, 1901	
*Clinostomum* cf. *marginatum* Rudolphi, 1819	Mesenteries
Diplostomidae Poirier, 1886	
*Uvulifer ambloplitis* (Hughes, 1927)	Skin
*Posthodiplostomum* cf. *minimum* (MacCallum, 1921)	Mesenteries
Nematoda	
Capillariidae Railliet, 1915	
*Freitascapillaria moraveci* Caspeta‐Mandujano, Salgado‐Maldonado and Vázquez, 2009	Gall bladder
Cystidicolidae Skrjabin, 1946	
*Spinitectus mexicanus* Caspeta‐Mandujano, Moravec and Salgado‐Maldonado, 2000	Intestine
Nematode larvae	
Dioctophymatidae Railliet, 1915	
*Eustrongylides* sp.	Mesenteries
Anisakidae Railliet and Henry, 1912	
*Contracaecum* sp.	Mesenteries
Rhabdochonidae Travassos, Artigas and Pereira, 1928	
*Rhabdochona* sp.	Intestine

**TABLE 3:** Parasite taxa infecting *Pseudoxiphophorus bimaculatus* collected in 2016 from 11 localities at the Río La Antigua basin, Veracruz, Mexico

Parasite taxa No. of host examined	Pixquiac 20	Xico 20	Agua Bendita 19	Teocelo 20	Baxtla 20	Jalcomulco 20
*U*. *vaginoclaustroides*	35/1 ± 1.6; 19/1.6		74/6.9 ± 7.0; 131/0.9	75/7.5 ± 8.3; 150/1.1	80/14.0 ± 23.0; 270/2.8	55/2.6 ± 4.1;52/2.1
*G*. *takoke*	5/0.1 ± 0.2; 1/0	30/0.4 ± 0.6; 7/−0.04	16/0.2 ± 0.4; 3/−0.7	30/0.6 ± 1;12/1.3	35/0.8 ± 1.2; 15/1.2	25/0.3 ± 0.6; 6/0.3
*G*. *xalapensis*	25/0.4 ± 0.7; 7/0.8		10/0.1 ± 0.3; 2/−0.5	35/1.1 ± 1.8; 21/2.1	35/0.8 ± 1.4; 16/1.6	5/0.1 ± 0.2; 1/0
*Gyrodactylus* sp.	50/1.2 ± 0.7; 23/0.5	15/0.4 ± 0.9; 7/3.3	21/0.2 ± 0.4; 4/−0.8	80/5.1 ± 4.1; 101/0.4	90/3.6 ± 1.8; 71/−0.02	15/0.2 ± 0.4; 3/−0.7
*P*. *inecoli*		25/0.9 ± 2.0; 17/4.4	26/1.3 ± 2.8; 25/3.6	40/1.5 ± 2.2; 29/1.6	20/1.5 ± 3.1; 29/3.8	15/1.0 ± 2.7; 20/6.0
*P*. *heterandriae*	5/0.7 ± 0.7; 1/0			10/0.2 ± 0.5; 3/3.9	15/0.2 ± 0.5; 4/1.8	45/4.4 ± 8.9; 88/3.8
*E*. *leopoldinae*						25/1.9 ± 6.7; 38/11.8
*C*. *formosanus*						85/38 ± 80; 750/4.5
*A*. (*Leighia*) *megalocephala*						5/0.2 ± 0.2:1/0
*A*. (*Phagicola*) *macrostoma*						
*C*. cf. *marginatum*						10/0.2 ± 0.5; 3/3.9
*U*. *ambloplitis*					5/0.2 ± 0.7;3/13.3	60/2.0 ± 2.1; 40/0.6
*P*. cf. *minimum*					5/0.1 ± 0.2; 1/0	45/2.0 ± 4.4;39/4.6
*S*. *mexicanus*			31/0.4 ± 0.7; 8/0.3	30/0.5 ± 1; 10/2		35/1.2 ± 1.9; 23/1.8
*F*. *moraveci*		5/0.1 ± 0.4; 2/10	79/1.9 ± 1.7; 36/0.3			60/3.0 ± 3.1; 57/0.7
*Eustrongyloides* sp.			5/0.1 ± 0.2; 1/0			
*Rhabdochona* sp.						
*Contracaecum* sp.						

Parasite taxa No. of hosts examined	Apazapan 20	Los Pescados 20	El Carrizal 20	Puente Nacional 21	Antigua Presa 20
*U*. *vaginoclaustroides*	65/2.5 ± 3.4; 50/1.5	25/0.4 ± 0.7; 7/0.8	10/0.3 ± 0.8; 5/5.8	25/0.3 ± 0.6; 6/0.4	25/0.9 ± 1.7; 17/2.7
*G*. *takoke*		20/0.3 ± 0.6; 5/0.8	5/0.1 ± 0.2; 1/0	10/0.1 ± 0.5; 3/4.2	5/0.1 ± 0.2; 1/0
*G*. *xalapensis*		10/0.1 ± 0.3; 2/−0.5	25/0.3 ± 0.6; 6/0.2	15/0.2 ± 0.5; 4/2.0	
*Gyrodactylus* sp.		35/0.6 ± 0.9; 12/0.8	10/0.1 ± 0.3; 2/−0.5	15/0.1 ± 0.4; 3/−1.0	
*P*. *inecoli*	10/0.2 ± 0.5; 3/3.9				
*P*. *heterandriae*	35/0.7 ± 1.1; 13/1.5				
*E*. *leopoldinae*					
*C*. *formosanus*	20/0.3 ± 0.7; 6/1.4		10/1.9 ± 8.3; 38/18.4	5/0.1 ± 0.2; 1/0	30/0.8 ± 1.5; 16/2.3
*A*. (*Leighia*) *megalocephala*	10/0.3 ± 0.9; 5/9.2				
*A*. (*Phagicola*) *macrostoma*	5/0.1 ± 0.2; 1/0				
*C*. cf. *marginatum*	5/0.1 ± 0.4; 2/10				
*U*. *ambloplitis*	5/0.1 ± 0.4; 2/10	5/0.1 ± 0.2; 1/0	5/0.2 ± 0.7; 3/13.3		
*P*. cf. *minimum*			5/0.2 ± 0.7; 3/13.3		5/0.1 ± 0.2; 1/0
*S*. *mexicanus*					
*F*. *moraveci*	56/0.9 ± 0.9; 17/0.02				
*Eustrongylides* sp.					
*Rhabdochona* sp.	5/0.1 ± 0.2; 1/0			5/0.1 ± 0.2; 1/0	
*Contracaecum* sp.					45/0.9 ± 1.2; 17/0.8

Note

Data are percent of infection/ and mean abundance ± *SD* of infections; total no. of helminth individuals collected/*J* (aggregation) values.

### 3.2 Species richness and abundance

The analysis of species accumulation curves (Table 4) allowed confidence that the number of fish examined from each locality was large enough to record the majority of helminth species from each locality. The slope of the last point of each curve calculated from Clench's model was small enough (≤0.1) to indicate an asymptotic shape of the curve; therefore, the observed number of helminth species from each component community was not less than 70% of the asymptotic value, that is, the real number of species in each component community, except for El Carrizal and Pixquiac, where only 41% and 65% of the helminth species were recovered, respectively (Table 4). Confirming the anterior trend, the values of the bootstrap nonparametric estimator suggested that we recorded most, if not all (78%–98%) of the helminth species from each locality (Table 4). Only a few rare helminth taxa were likely to have been missed because of the number of hosts examined. Thus, analyses allowed us to examine almost the entire composition of the helminth communities parasitizing populations of *P*. *bimaculatus* along the La Antigua River basin. Therefore, patterns derived from the repeatability of community structure, species saturation, and species co‐occurrence are based on the helminth species that were most characteristic in structuring the community.

**TABLE 4:** Summary of the richness analysis and parameters of the cumulative species curves of species for 11 component communities of helminths of *Pseudoxiphophorus bimaculatus* from the La Antigua river basin, Veracruz, Mexico (in all cases the correlation coefficient *R* ^2^ between the observed data and Clench's model > .97)

Locality	No. of hosts examined	S Observed	Clench's model parameters		Richness (Clench *a*/*b*)	Slope (Clench *a*/(1 + b*n)^2^	Bootstrap estimator
			*a*	*b*			
Pixquiac	20	5	0.36	0.06	6	0.074	5.72
Teocelo	20	7	5.44	0.71	7.66	0.023	7.12
Baxtla	20	8	3.38	0.38	8.89	0.045	9.72
Xico	20	5	0.93	0.14	6.64	0.064	5.75
Agua Bendita	19	8	3.33	0.36	9.25	0.054	8.53
El Carrizal	20	8	0.77	0.04	19.25	0.23	9.80
Río de los Pescados	20	5	1.23	0.29	4.24	0.02	5.49
Jalcomulco	20	14	6.88	0.44	15.63	0.07	14.92
Apazapán	20	10	1.94	0.15	12.93	0.12	11.68
Puente Nacional	21	6	0.83	0.09	9.22	0.10	7.64
Antigua Presa	20	5	1.31	0.22	5.95	0.44	5.72

Fish size in the different localities ranged from 30 mm to 100 mm total length (mean length of the 220 fish was 52.7 ± 13.4 *SD* mm). This variation was significant when comparing fish sizes between localities (*F* = 6.1, *p* < .001). Tukey's test showed that smaller fish were found in the Antigua Presa and Apazapan locations (Appendix S2: Figure A1). However, the size class of fish remained consistent in each locality. Furthermore, neither helminth species richness nor abundance (total number of helminth individuals, monogeneans, or adult endohelminths, separately) correlated with the mean size of the fish examined in each locality (Appendix S2: Figures A2 and A3).

### 3.3 Unsaturation of communities

**FIGURE 2:**
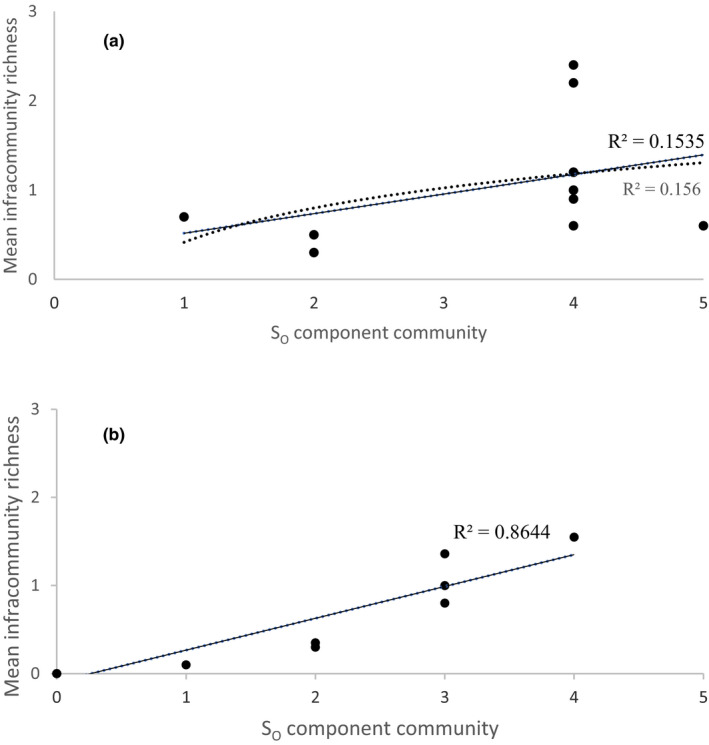
Relationship between component community species richness (*S*_O_) and mean infracommunity species richness; (a) monogeneans; (b) endohelminths

We did not find a curvilinear relationship between mean richness recorded in an infracommunity and component community richness for monogeneans or endohelminths (Figure 2). For monogeneans, the proportion of variance in the distribution of observations that explained a curvilinear relationship was the same as that which explained a linear relationship (*r* ^2^ = .15). For endohelminths, a high proportion of variance in the distribution of observations was explained by a linear relationship (*r* ^2^ = .86). Thus, we did not find an upper limit of local species richness in the individual hosts in relation to the size of the regional pool of species.

The maximum richness of the infracommunities (in seven cases for the monogeneans and two cases for the endohelminths) was below that of the component communities (Table A1). However, we found a weak positive correlation between the observed richness of monogeneans (*S*_OM_) in the component community and the mean richness of monogeneans of the infracommunities (*r* = .38), as well as a very weak and negative correlation with the maximum richness recorded in an infracommunity (*r* = −.13), that is, increasing the monogenean richness of the component communities did not signify more species in the infracommunities. No correlation was found between the total number of individual monogeneans in the component community versus the observed richness of species of monogeneans in the component communities (*r* = .08); therefore, populations of monogeneans may increase independently of richness. The maximum infracommunity richness in our study was limited only by the availability of species in the component communities.

We found a positive and very strong correlation between the observed richness of endohelminths (*S*_OE_) based on two trematode (*Paracreptotrematoides heterandriae* and *Phyllodistomum inecoli*) and two nematode species (*Freitascapillaria moraveci* and *Spinitectus mexicanus*) in the component community and the mean richness of the endohelminths in the infracommunities (*r* = .95**). The correlation between the maximum richness recorded in an infracommunity was strong but not significant (*r* = .73 *p* = .07), suggesting, at least partially for endohelminths, that as the richness of the component communities increased, there were more species in the infracommunities. Our data also showed that the increase in the individual endohelminth species correlated positively and strongly with species richness (*r* = .91***) and that mean endohelminth species richness in infracommunities increased with the total endohelminth individuals recorded in the component community (*r* = .93***). These data suggest that the richness of endohelminths was density‐dependent.

### 3.4 Intraspecific aggregation of helminths

**FIGURE 3:**
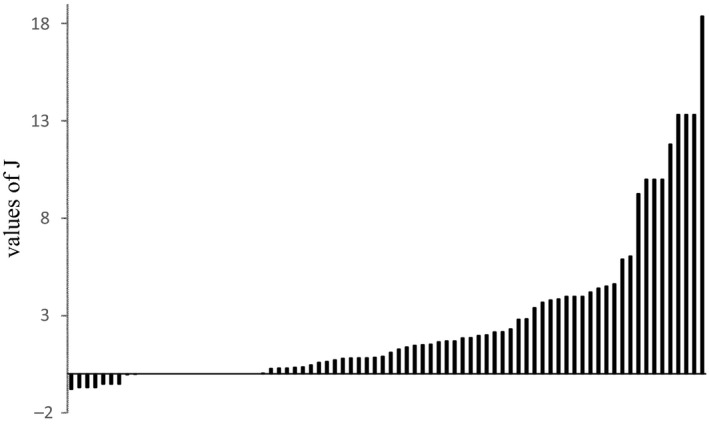
Intraspecific aggregations. Eighty values of *J* calculated for each parasite species. Note 56 values *J* > 0 (range: 0.02–18.39), 9 values *J* < 0 (range: −0.79 to −0.01), and 15 values *J* = 0

Most (53/80) of the calculated *J* values were positive (Table 3, Figure 3; range: 0.02–18.39). All endohelminth records showed aggregation. Species found in low numbers did not show aggregation (*J* values = 0 corresponded to records of a single parasitic specimen; Table 3). Nine values of *J* < 0 belonged to a low number of infections by monogeneans (i.e., two to seven monogenean individuals distributed in approximately the same number of hosts, with *J* values ranging from −0.79 to −0.42).

*J* values for the three *Gyrodactylus* species suggested they were density‐dependent. We identified significant and positive correlations between *J* values and the mean intensity of each species in the component communities where they were recorded (*G*. *xalapensis* *r* = .95***, *G*. *takoke* *r* = .85**, *Gyrodactylus* sp. *r* = .65*). A very strong positive correlation between *J* values and the mean intensity of the invasive metacercariae *C*. *formosanus* was also found, *r* = .90**. This pattern of density dependence was not found in any of the other helminths studied.

### 3.5 Interspecific aggregation of helminths

A high proportion of interspecific association index *C*_1,2_ values were <0, indicating between‐species segregation (i.e., a high proportion of the analyzed species pairs were negatively correlated). We calculated 77 values of interspecific aggregation between 16 pairs of species including all the registered monogeneans, *C*. *formosanus,* and the endohelminth taxa in each location (Table 5). Seventy‐seven % (47/61) of associations involving ectoparasitic monogeneans and metacercariae of *C*. *formosanus* were negative (*C*_1,2_ < 0; Table 5). Calculated values of *C*_1,2_ in these cases suggested the presence of two species in the same component community and co‐infections in a few infracommunities. Inclusion of *C*. *formosanus* in these calculations could be justified because these parasites encyst in the gills, alter tissues, and occupy space, thus potentially interacting negatively with monogeneans.

**TABLE 5:** Number of positive and negative interspecific aggregation values C_1,2_ (±) within fish infected by both species of helminth pairs. Below the diagonal are values of *A*_1,2_ > 1 within fish infected by both species of helminth pairs. A. Ectoparasitic monogeneans and metacercariae of *C*. *formosanus*, B. Endohelminths

A. Monogeneans and *C*. *formosanus*					
	*U*. *vaginoclaustroides*	*G*. *xalapensis*	*G*. *takoke*	*Gyrodactylus* sp.	*C*. *formosanus*
*U*. *vaginoclaustroides*		0/8	2/6	1/8	0/4
*G*. *xalapensis*	3		3/5	4/4	0/2
*G*. *takoke*	4	3		4/5	0/2
*Gyrodactylus* sp.	4	4	3		0/3
*C*. *formosanus*	4	1	1	1	

B. Endohelminths				
	*P*. *inecoli*	*P*. *heterandriae*	*F*. *moraveci*	*S*. *mexicanus*
*P*. *inecoli*		½	2/2	0/3
*P*. *heterandriae*	1		0/2	1/1
*F*. *moraveci*	4	2		1/1
*S*. *mexicanus*	3	2	2	

Interspecific aggregation *C*_1,2_ values were positively correlated with richness and abundance parameters. Considering the 61 calculated values of *C*_1,2_ for ectoparasites, monogeneans, and *C. formosanus*, we found moderate, significant positive correlations when regressed against the mean number of species per host (*r* = .49***), the maximum number of species registered in an infracommunity (*r* = .56***), the total number of individual monogeneans in the component community (*r* = .53***), the mean number of individual monogeneans per host (*r* = .52***), and the maximum number of monogeneans recorded in a host (*r* = .55***). Density dependence of *C*_1,2_ values was evident when each species pair of monogeneans was examined separately. From the analysis of all possible correlations of abundance and monogenean richness in the six possible pairs of monogenean species (Table 5), we found density dependence in four of the pairs (Table 6). The species pairs *G*. *xalapensis*/*G*. *takoke* and *G*. *xalapensis*/*Gyrodactylus* sp. were recorded from each of the eight component communities; however, their calculated *C*_1,2_ values did not correlate with any of the richness or abundance parameters.

**TABLE 6:** Spearman's rank correlation coefficients obtained when comparing *C*_1,2_ values versus several density parameters of four monogenean species pairs

	No. of component communities in which it was recorded	Spearman's rank correlation between *C*_1,2_ values and:
*G*. *takoke*/*Gyrodactylus* sp.	9	Maximum richness of monogenean species per host *r* = .76**
		Maximum no. of monogeneans in an infracommunity *r* = .68*
		Total # of *G*. *takoke* *r* = .95***
*U*. *vaginoclaustroides*/*Gyrodactylus* sp.	9	Maximum richness of monogenean species per host *r* = .67*
		Mean richness of monogenean species per host *r* = .83**
		Maximum no. of monogeneans in an infracommunity *r* = .72*
		Total no. of monogeneans in the component community *r* = .74*
		Mean no. of monogeneans per host *r* = .72*
		Total # of *Gyrodactylus* sp. *r* = .84*
*U*. *vaginoclaustroides*/*G*. *takoke*	8	Maximum richness of monogenean species per host *r* = .96***
		Total no. of monogeneans in the component community *r* = .82**
		Mean no. of monogeneans per host *r* = .83**
		Maximum no. of monogeneans in an infracommunity *r* = .79*
		Total # of *U*. *vaginoclaustroides* *r* = .79*
		Total # of *G*. *takoke* *r* = .97***
*U*. *vaginoclaustroides*/*G*. *xalapensis*	8	Maximum richness of monogenean species per host *r* = .82*
		Mean richness of monogenean species per host *r* = .81*
		Total no. of monogeneans in the component community *r* = .84*
		Maximum no. of monogeneans in an infracommunity *r* = .85*
		Mean no. of monogeneans per host *r* = .85*

Note

The species pairs *G*. *xalapensis*/*G*. *takoke* and *G*. *xalapensis*/ *yrodactylus* sp. were each recorded from eight component communities; however, any correlation between their values of C_1,2_ and the richness density parameters were recorded.

* *p* < .05.

** *p* < .01.

*** *p* < .001.

Most (11/16, 68%) interactions between the four species of endohelminths, the two trematodes *Paracreptotrematoides heterandriae* and *Phyllodistomum inecoli*, and the two nematodes *Freitascapillaria moraveci* and *Spinitectus mexicanus*, were negative (value of *C*_1,2_ < 0), while 32% (5/16) had positive *C*_1,2_ values (Table 5). Coinfections of endohelminth taxa were recorded in one to five infracommunities. *C*_1,2_ values calculated for endohelminths were not density‐dependent, that is, they were not correlated with richness and abundance parameters in either component communities or infracommunities.

### 3.6 Association between pairs of parasite species

Associations among monogeneans were consistently recorded in several locations. A total of 78% (48/61) of the calculated correlations between 61 pairs of ectoparasitic monogeneans and *C*. *formosanus* metacercariae were negative. Of the 12 pairs of endohelminths in the component communities, 83% (10/12) had negative correlations in the intensity of species; 10 of these comparisons for the ectoparasites and five for the endoparasites were significant (Table 7). Significant negative interactions were detected between the three species pairs of monogeneans, *Urocleidoides vaginoclaustroides*/*G*. *xalapensis*, *U*. *vaginoclaustroides*/*G*. *takoke*, and *U*. *vaginoclaustroides*/*Gyrodactylus* sp., and between *U*. *vaginoclaustroides*/*C*. *formosanus*, which were repeated in more than one system (Table 7). However, only one species pair of endohelminths, *Phyllodistomum inecoli*/*Paracreptotrematoides heterandriae* showed significant negative interactions in more than one system (Table 8). Only one significant positive interaction was found (i.e., *P*. *inecoli*/ *F*. *moraveci* in Agua Bendita r = 0.54*). No correlation was found between the abundance of any of the species listed above with the size of the hosts (total length) when component communities were analyzed (Tables 7 and 8).

**TABLE 7:** Matrix of pairwise associations (Spearman's rank correlation coefficients) between the intensity of infection of ectohelminth parasites of *P*. *bimaculatus* from 11 localities of La Antigua River basin, Veracruz, Mexico

	*U*. *vaginoclaustroide*	*G*. *takoke*	*G*. *xalapensis*	*Gyrodactylus* sp.	*C*. *formosanus*
Pixquiac					
*U*. *vaginoclaustroides*		−0.58	−0.86***	−0.48	
*G*. *takoke*	8		−0.70	−0.57*	
*G*. *xalapensis*	12	6		0.24	
*Gyrodactylus* sp.	13	11	12		
Xico					
*G*. *takoke*				0.21	
*Gyrodactylus* sp.		7			
Agua Bendita					
*U*. *vaginoclaustroides*		−0.47*	−0.20	−0.03	
*G*. *takoke*	16		−1.00	−0.70	
*G*. *xalapensis*	15	5		−0.25	
*Gyrodactylus* sp.	14	6	6		
Teocelo					
*U*. *vaginoclaustroides*		−0.18	−0.005	0.26	
*G*. *takoke*	16		0.59	0.02	
*G*. *xalapensis*	15	9		−0.09	
*Gyrodactylus* sp.	18	18	18		
Baxtla					
*U*. *vaginoclaustroides*		−0.21	−0.18	−0.27	
*G*. *takoke*	17		−0.32	0.36	
*G*. *xalapensis*	18	9		0.28	
*Gyrodactylus* sp.	18	19	19		
Jalcomulco					
*U*. *vaginoclaustroides*		−0.22	−0.48	−0.72**	−0.60**
*G*. *takoke*	13		−0.25	0	−0.49
*G*. *xalapensis*	12	5		0	0.12
*Gyrodactylus* sp	14	6	3		−0.23
*C*. *formosanus*	19	19	17	18	
Apazapan					
*U*. *vaginoclaustroides*					−0.54*
*C*. *formosanus*	15				
Río de los Pescados					
*U*. *vaginoclaustroides*		−0.81	−0.66	−0.70**	
*G*. *takoke*	7		0	−0.08	
*G*. *xalapensis*	8	5		−0.66	
*Gyrodactylus* sp	11	8	8		
El Carrizal					
*U*. *vaginoclaustroides*		−0.86	−0.86**	−0.94	
*G*. *takoke*	3		−0.77	−1.00	
*G*. *xalapensis*	7	6		0	
*Gyrodactylus* sp	4	3	6		
Puente Nacional					
*U*. *vaginoclaustroides*		−0.86*	−0.90**	−0.72	−0.25
*G*. *takoke*	7		0.33	−0.96	−0.86
*G*. *xalapensis*	8	4		−0.94	−0.81
*Gyrodactylus* sp	7	5	5		−1.00
*C*. *formosanus*	5	3	3	3	
Antigua Presa					
*U*. *vaginoclaustroides*				−0.66	−0.22
*Gyrodactylus* sp	6				−0.61
*C*. *formosanus*	8			7	

Note

Fish not harboring worms from either species in a pairwise association (double zeros) were excluded; actual sample sizes are the numbers of fish harboring at least one of the two species in a pair and are given below the diagonal.

* *p* < .05.

** *p* < .01.

*** *p* < .001.

**TABLE 8:** Matrix of pairwise associations (Spearman's rank correlation coefficients) between the intensity of infection of endohelminth parasites of *P*. *bimaculatus* from four localities of La Antigua River basin, Veracruz, Mexico

	*P*. *inecoli*	*P*. *heterandriae*	*F*. *moraveci*	*S*. *mexicanus*
Agua Bendita				
*P*. *inecoli*			0.54*	−0.86**
*F*. *moraveci*	15			0.10
*S*. *mexicanus*	8		17	
Teocelo				
*P*. *inecoli*		−0.46		−0.51
*P*. *heterandriae*	9			−0.48
*S*. *mexicanus*		6		
Baxtla				
*P*. *inecoli*		−0.87*		
*P*. *heterandriae*	7			
Jalcomulco				
*P*. *inecoli*		0.24	−0.37	−0.39
*P*. *heterandriae*	9		−0.58**	−0.08
*F*. *moraveci*	14	17		−0.54**
*S*. *mexicanus*	8	10	16	
Apazapan				
*P*. *inecoli*		−0.75*	−0.44	
*P*. *heterandriae*	9		−0.38	
*F*. *moraveci*	13	15		

Note

Fish not harboring worms from either species in a pairwise association (double zeros) were excluded; actual sample sizes are the numbers of fish harboring at least one of the two species in a pair and are given below the diagonal.

* *p* < .05.

** *p* < .01.

*** *p* < .001.

### 3.7 Decrease in competition

**FIGURE 4:**
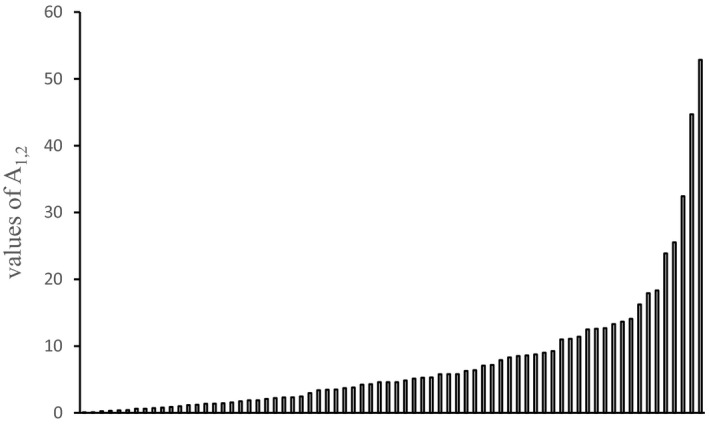
Interspecific aggregations. Seventy‐seven values of *A*_1,2_ calculated for each of 16 pair of parasite species. Note 64 values *A*_1,2_ > 1 (range: 1.1–52.8)

Most (42/77) *A*_1,2_ values calculated between the 16 pairs of species, including all monogeneans, *C*. *formosanus*, and endohelminth taxa in each location were >1 (Table 5, Figure 4). Therefore, intraspecific aggregations were stronger than interspecific aggregations. No correlation was found between the values of *A*_1,2_ and richness parameters (number of observed species in the community component, and mean and maximum observed species per host) or with abundance parameters (total number of helminths recorded, and mean and maximum number of helminths per infracommunity). Therefore, the increase in diversity or abundance did not correlate with an increase in intraspecific aggregation compared with interspecific aggregation.

## 4 DISCUSSION

### 4.1 Low diversity of helminth communities

Our results show that interspecific interactions play an important role in structuring the low‐diversity helminth communities of a tropical freshwater fish and that interspecific competition can occur in species‐poor, nonsaturated communities. Aggregation is an important factor for determining the local richness of parasites in fish populations. Intraspecific aggregation allows the coexistence of species in the same host population by decreasing the overall competition intensity.

Low species richness and abundance of helminths were most evident in our study system. For endohelminth communities, we recorded a mean species richness between 0.1 ± 0.2 and 1.55 ± 1.05 species among the 11 sampled localities, and a mean infracommunity abundance between 0.1 ± 0.2 and 9.4 ± 9.8 for the total individuals. These are relatively low numbers compared with values for intestinal infracommunities of other tropical or subtropical fish species such as the cichlids *C. pearsei* (3.6 ± 0.7 species; 353 ± 27 individuals, Pineda‐López, 1994), *C*. *urophthalmus* (2.2 ± 0.65 species; 76.8 ± 66.0 individuals, Salgado‐Maldonado & Kennedy, 1997), and *C. synspilum* (2.4 ± 1.2 species; 34 ± 52 individuals, Vidal‐Martínez & Kennedy, 2000).

Little data are available for comparisons of ectoparasite richness and density in freshwater fishes. Bellay, Takemoto, and Oliveira (2012) counted 6,650 individual monogeneans from 13 taxa in 61 specimens of the piranha *Serrasalmus marginatus* from the Paraná River, Brazil. The mean number of monogeneans ranged from 64.4 to 156.9 individuals per fish. Agrawal et al. (2017) counted 10,920 individual monogeneans of five species of *Thaparacleidus* parasites in 72 specimens of the Indian freshwater shark *Wallago attu*, with a mean of 151.6 monogeneans per examined fish. Our numbers are comparatively low (1,048 monogeneans of four species from 220 examined hosts, with a mean between 1.45 ± 0.93 and 18.6 ± 23 monogeneans per infected host).

### 4.2 Nonsaturated communities

No limitation was found in the number of species for either monogenean or endohelminths in the infracommunities, which agrees with findings from previous studies (Morand et al., 1999; Rohde, Hayward, & Heap, 1995; Salgado‐Maldonado et al., 2019). Therefore, infracommunities were not saturated by local residents; rather, infracommunity richness (local richness) was dependent on the size of the species pool of the component community (regional richness). Two additional observations pointed toward nonsaturation. First, empty niches were observed because the maximum richness of the infracommunities was lower than that the component community. Therefore, maximum potential infracommunity richness was less likely in the studied communities, as would be expected if interspecific interactions among parasites were important and led to species saturation. Second, evidence was found that increased monogenean richness of component communities did not signify more species in the infracommunities. The proportional relationship between endohelminth richness in the component community and richness in the infracommunities also suggests that a maximum level of richness did not exist, which was consistent with the absence of saturation in the endohelminth communities. The tendency toward nonsaturation in infracommunities was more obvious for the endohelminths than for the monogeneans; therefore, species interactions might be negligible. Rohde (1991) suggested that most gill parasite species live in low‐density populations in resource‐rich habitats and that sections of available niches for ectoparasites remain empty. However, an alternative explanation for our observations is that infracommunities appeared nonsaturated owing to species exclusion following interspecific interactions.

### 4.3 Interspecific relationships

Contrary to the expectations for impoverished, low‐density, nonsaturated communities, our results on species associations (expressed as negative associations between pairs of helminth species and the *C*_1,2_ index of interspecific aggregation values) provide overall support for the role of negative, probably competitive, interactions in shaping helminth communities; especially among monogeneans. We found consistent, although not always significant, negative correlations between the numbers of helminth pairs of helminth species. Five of the species pairs of monogeneans and one species pair of endohelminths yielded a significant negative correlation in more than one location.

Consistent negative interactions are strong evidence of competitive interactions between species (Dezfuli et al., 2001; Poulin, 2001, 2007; Poulin & Valtonen, 2002). We contend that these are not spurious covariances for three main reasons. First, most statistical methods that are used to detect species covariances are more sensitive to positive associations than they are to negative ones (Haukisalmi & Henttonen, 1993). Second, our data include more common species with high prevalence, which could lead to a high number of positive associations (Lotz & Font, 1994). Third, we did not include rare species recorded in the component community, which could have produced spurious negative associations (Lotz & Font, 1994). The role of host size as a potential confounding factor creating spurious covariances can also be dismissed, because our results showed that the number of monogeneans in each species pair that exhibited significant negative correlations was not correlated with the size of *P*. *bimaculatus* examined at any given site. Therefore, the recorded negative covariances were independent of the possible accumulation of monogeneans in a larger host.

The number of negative covariances we found was notable because variance tests on binary presence–absence data for parasitic species in infracommunities (Schluter, 1984) indicate that the number of positive covariances equal the number of negative covariances if infracommunities are random assemblages, which was assumed in the present study as a null model when testing for pairwise associations (see also Poulin, 2005). Thus, we assumed that the high number of negative correlations we recorded was indicative of the role of negative, probably competitive, interactions in shaping the helminth communities. However, the observed patterns of species associations must be tested against other adequate null models in future studies (Lotz & Font, 1994; Poulin, 1997; Simberloff, 1990; Simberloff & Moore, 1997).

We reported a high proportion of values of interspecific association index *C*_1,2_ < 0, indicating that there was segregation and that a high proportion of the 16 pairs of species analyzed were negatively correlated in our communities. Given that the *C*_1,2_ index is a measure of the proportional increase in the number of heterospecific helminth competitors regarding a random association (Ives, 1988, 1991), both the *C*_1,2_ < 0 values and negative correlations between the actual parasite numbers of pairs of species are strong evidence for the occurrence of interactions in the communities studied. Therefore, the present results support previous conclusions by Kennedy (1985, 1992) and Vidal‐Martínez and Kennedy (2000) that interspecific competition can occur in species‐poor, isolationist, and nonsaturated communities. Interspecific competition, and thus its detectability, may vary among locations with the abundance of species because the prevalence and intensity of infection affect the magnitude and direction of pairwise associations as well as their detectability (Lotz & Font, 1994; Poulin & Valtonen, 2002).

The observed negative associations of the species pairs of monogeneans in the communities might be caused by the transmission of monogeneans in clumps from fish to fish, which could lead to a transfer of associations (Dezfuli et al., 2001; Lotz, Bush, & Font, 1995). This was noted recently in a different host–parasite system, suggesting it could be a general pattern (Salgado‐Maldonado et al., 2019). Therefore, associations between species could be transferred from the existing associations by passive transportation of monogeneans from fish to fish. However, this also potentially highlights the role of competition in the monogenean community structure with interspecific interactions occurring in the actual fish host. When monogeneans effectively disperse and colonize free patches, they compete with one another (Ives, 1988; Slatkin, 1974). Given that we recorded a high consistency in the distribution of individuals of different species, we assumed that the transmission of some species of monogeneans may be combined so that the colonization of new fish within a component or between components faces the problem of the simultaneous arrival of two or more heterospecific individuals. Simultaneous spreading from a common source and joint colonization by heterospecific parasite species could imply that their “exported” interspecific interaction could contribute to structure the resulting, new communities. This is because when the transmission of propagules is multiple or linked, these species will have to compete even at low population densities (Ives, 1988).

We propose that the negative association recorded in two different locations for the endohelminth adult trematodes, the Gorgoderidae *Phyllodistomum inecoli* (from the urinary bladder) and the Allocreadiidae *Paracreptotrematoides heterandriae* (from the intestine), reflects the interactions among metacercariae in intermediate hosts and might have nothing to do with species interactions operating in *P. bimaculatus*, the definitive host. Considering the general biology of the families, both these trematodes might infect *P. bimaculatus* similarly. Therefore, the recruitment of one species may not be independent of the other species. Both these families display a three host or abbreviated life cycle. The first intermediate hosts are usually bivalves (clams of the genera *Pisidium*, *Sphaeridium*, and *Musculium*), while metacercariae generally encyst in damselflies, trichopteran, or chironomid larvae or the larvae of diving beetles (Yamaguti, 1975). The definitive host *P. bimaculatus* becomes infected after ingesting infected intermediate hosts. Thus, the observed association among these trematodes might have originated when the fish preyed on an intermediate host, which may have contained larvae of more than one helminth species (Bush, Heard, & Overstreet, 1993; Lotz et al., 1995). This structure of larval helminth communities can then be transferred to adult helminth communities (Poulin, 2001).

Our results concerning endohelminths agreed with previous studies that found that pairwise associations between gastrointestinal species of helminths of freshwater fishes were erratic and unpredictable, including studies on *Salmo trutta* in Italy (Dezfuli et al., 2001), *Perca fluviatilis* and *Rutilus rutilus* in Finland (Poulin & Valtonen, 2002), marine fish species *Epinephelus morio*, and the freshwater *Cichlasoma urophthalmus* in Mexico (Vidal‐Martínez & Poulin, 2003). No pairwise association was observed consistently among the localities sampled, and random patterns in the structure of parasite communities were observed only sporadically (Dezfuli et al., 2001; Poulin & Valtonen, 2002; Vidal‐Martínez & Poulin, 2003). Local factors or short‐term influences could mask or eliminate any competitive interaction.

### 4.4 Intra‐ and interspecific aggregation

Both monogeneans and endohelminths showed high population aggregation. A fundamental difference between them is that the interspecific association *C*_1,2_ values increased with monogenean richness and number of individuals, whereas the aggregation of endohelminths did not show this density dependence. Therefore, intraspecific aggregation could have distinct origins in both subgroups. We recorded higher intraspecific aggregation rates than interspecific aggregation rates in both subgroups, which could facilitate species coexistence. The extent to which intraspecific aggregation will be high enough for interspecific aggregation to be important for coexistence can only be determined with planned experiments on particular communities (Ives, 1988). However, these communities can only be fully understood by examining how new helminths are recruited, and improved knowledge regarding the biology of helminth species, including modes of transmission and host infection, and experimental studies are urgently required.

In conclusion, although based on species‐poor, nonsaturated communities with vacant niches, our study documented numerical effects elicited by the presence of one helminth species on the abundance of another species, especially between monogeneans. This would suggest that interspecific competition is likely to occur in isolationist communities. Our data provide empirical evidence that high aggregation levels of these helminths contribute to species richness within a population of hosts because intraspecific and interspecific aggregations would facilitate contact between individual parasites and the coexistence of the most frequent species.

## CONFLICT OF INTEREST

None.

## AUTHOR CONTRIBUTION

**Guillermo Salgado‐Maldonado:** Conceptualization (lead); Data curation (lead); Formal analysis (lead); Funding acquisition (lead); Investigation (lead); Methodology (lead); Project administration (lead); Resources (lead); Supervision (lead); Validation (lead); Writing‐original draft (lead); Writing‐review & editing (lead). **Juan Manuel Caspeta‐Mandujano:** Conceptualization (supporting); Data curation (supporting); Investigation (supporting); Methodology (supporting); Validation (supporting); Writing‐original draft (supporting); Writing‐review & editing (supporting). **Edgar F. Mendoza‐Franco:** Conceptualization (supporting); Data curation (supporting); Investigation (supporting); Methodology (supporting); Validation (supporting); Writing‐original draft (supporting); Writing‐review & editing (supporting). **Miguel Rubio‐Godoy:** Data curation (supporting); Investigation (supporting); Methodology (supporting); Validation (supporting); Writing‐original draft (supporting). **Adriana García‐Vásquez:** Data curation (supporting); Investigation (supporting); Validation (supporting). **Norman Mercado‐Silva:** Conceptualization (supporting); Data curation (supporting); Methodology (supporting); Writing‐original draft (supporting). **Ismael Guzmán‐Valdivieso:** Data curation (supporting); Methodology (supporting). **Wilfredo A. Matamoros:** Formal analysis (supporting); Writing‐original draft (supporting); Writing‐review & editing (supporting).

## Supporting information

Appendix S1

Appendix S2

## Data Availability

Data supporting the results will be archived in Data in Brief manuscript number DIB‐D‐20‐01261.
