# Supplementary material for: Competition from sea to mountain: Interactions and aggregation in low‐diversity monogenean and endohelminth communities in twospot livebearer Pseudoxiphophorus bimaculatus (Teleostei: Poeciliidae) populations in a neotropical river
Source: Ecol Evol. 2020 Aug 12;10(17):9115–31. doi: 10.1002/ece3.6557 (PMC7487236; doi:10.1002/ece3.6557)
Supplement: Supplementary file 2 — Appendix S2 [file ECE3-10-9115-s002.docx]

Appendix 2.

Size ranges of the fish examined for the different localities.

Fig. A1. Variation of total length (mm) of the fish examined for the different localities.


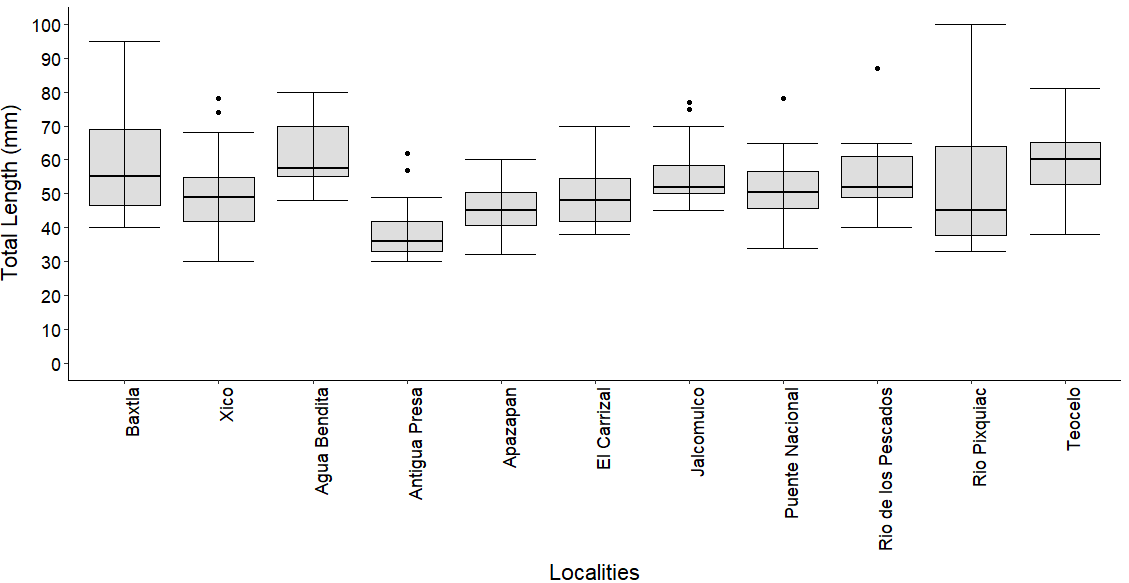


Correlations of richness and abundance with fish size

Fig. A2. Correlation between observed species richness and the mean total length of fish from each locality.

Fig. A3. Correlation between total number of helminth individuals and the mean total length of fish from each locality.
